# Health behaviours in 131,182 UK women planning pregnancy

**DOI:** 10.1186/s12884-021-04007-w

**Published:** 2021-07-28

**Authors:** Beth McDougall, Kimberley Kavanagh, Judith Stephenson, Lucilla Poston, Angela C. Flynn, Sara L. White

**Affiliations:** 1 Department of Mathematics and Statistics, University of Strathclyde, Glasgow, UK; 2 EGA Institute for Women’s Health, University College London, 74 Huntley Street, WC1E 6AU London, UK; 3 Department of Women and Children’s Health, School of Life Course Sciences, King’s College London, 10th Floor North Wing, St Thomas’ Hospital, Westminster Bridge Road, SE1 7EH London, UK; 4 Department of Nutritional Sciences, Franklin-Wilkins Building, 150 Stamford Street, London, SE1 9NH UK

**Keywords:** Preconception, Planning for pregnancy, Contraception, Pregnancy

## Abstract

**Background:**

A woman’s health at the time of conception lays the foundation for a healthy pregnancy and the lifelong health of her child. We investigated the health behaviours of UK women planning pregnancy.

**Methods:**

We analysed survey data from the ‘Planning for Pregnancy’ online tool (Tommy’s, UK). We described all women planning pregnancy and compared the frequency of non-adherence to preconception recommendations in women who had already stopped contraception (active planners) and those who had not (non-active planners).

**Results:**

One hundred thirty-one thousand one hundred eighty-two women from across the UK were included, of whom 64.8% were actively planning pregnancy. Of the whole cohort, twenty percent were smokers and less than one third took folic acid supplements (31.5%). Forty two percent engaged in less than the recommended 150 min of weekly physical activity and only 53.3% consumed five portions of fruit or vegetables 4 days a week. Smokers were 1.87 times more likely to be active planners than non-smokers (95% CI 1.79–1.94), and women who took folic acid were 7 times more likely to be active planners (95% CI 6.97–7.59) compared to women who did not. Smoking, drug use and lack of folic acid supplementation were common in younger women and those who were underweight.

**Conclusions:**

This unique survey of UK women has identified poor adherence to preconception recommendations in those planning pregnancies and supports the need for a greater public health focus on preconception health. This study provides a contemporary basis from which to inform preconception health advice and a benchmark to measure changes over time.

## Background

Health behaviours before pregnancy may profoundly influence pregnancy outcome, and also the future health of mother and child [1], as detailed in a recent Lancet series [2]. Unhealthy and risky behaviours may influence early embryonic viability or development, or if persistent after conception, the health of the fetus, and are difficult to modify once pregnancy is established [3, 4]. Public Health England (PHE) recommendations prior to pregnancy include maintaining a healthy diet, participating in frequent physical activity, taking folic acid supplements, encouraging emotional wellbeing, and ensuring cervical screening, sexual health tests and vaccinations are up to date [4]. High risk behaviours in the preconception period include smoking, alcohol consumption and illicit substance use. Obesity is also a risk, being associated with infertility, suboptimal embryonic health and complications in pregnancy [4].

Despite proposals for health policies that increase awareness of the importance of preconception health, there is a lack of knowledge among, and resources available, to women and their health care providers, particularly in primary care [2, 5–7]. A systematic review of healthcare providers’ views on preconception care provision suggested inadequacy, with provision of more barriers than facilitators [5]. In the Lancet series, the UK Preconception Partnership proposed a preconception health strategy with targeted public health policies and identification of women planning a pregnancy who would benefit from support, while incorporating the preconception period in existing strategies to counteract obesity and smoking. The Partnership also recommended that the UK adopt an annual report card evaluating metrics from various data sources which holds the government and relative authorities to account for the delivery of preconception advice and interventions [2].

Research has been conducted on the uptake of preconception care [8, 9], and studies are beginning to emerge on the effectiveness of intervention programmes [10–13]. In the UK, as in most developed countries, the metrics detailing women’s health behaviours prior to conception remain largely conjectural. Most previous studies on preconception health behaviours are retrospective in design, asking pregnant women to look back to their behaviours before conceiving, and therefore being subject to recall bias [3]. Alternatively, inferences of preconception health are drawn from national datasets of women of reproductive age [14], irrespective of pregnancy intention. We aimed to examine the health behaviours and lifestyle factors of a large prospective cohort of UK women planning a pregnancy who completed a digital pregnancy planning health tool. The tool was accessed on the website of a charitable organisation with extensive national and international reach (Tommy’s, UK). The study was designed in response to the call for a set of core metrics with which to monitor behavioural changes as a result of public health intervention, and thereby to contribute to the preconception report card as proposed by the Preconception Partnership.

## Methods

This study used data collected from women planning a pregnancy who used an on-line digital health tool (Tommy’s ‘Planning for Pregnancy’ tool; https://www.tommys.org/pregnancy-information/planning-pregnancy/planning-for-pregnancy-tool) between the 22nd of June 2018 and the 31st of August 2019. The online tool (a questionnaire) is designed to identify women at high risk of pregnancy complications, and to provide tailored advice to improve health and pregnancy outcomes. The questionnaire includes closed-ended questions designed to capture pregnancy planning and health. With support from PHE and the Royal College of Obstetricians and Gynaecologists (RCOG), it was developed by Tommy’s in consultation with multidisciplinary health and medical experts, and with women from the general public. Consumer testing was performed before going live.

### Data collection

The Tommy’s tool is openly accessible on the internet. In order to increase awareness and use, social media advertisement via Facebook was used to target women planning pregnancy and the resultant sample of women was self-selected. This included mainstream targeting (females, age 16–45), women from minority ethnic groups, low income women and those with a high BMI. Only data from women living in the UK were included in the analysis. To establish stage of pregnancy planning, women were asked if they had already stopped contraception or when they planned to stop. For the purposes of this study, and to assess preparedness for pregnancy, women who had already stopped contraception were defined as ‘active planners’ and those who had not stopped as ‘non‐active planners’. ‘Active planners’ were further divided into those who had stopped contraception less than or more than a year ago.

Data collected on health behaviours included smoking (current, yes/no), alcohol and caffeine consumption, illicit or recreational drug use, supplementation with folic acid (yes/no), consumption of 5 portions of fruit and vegetables at least 4 days a week (yes/no/don’t know) and weekly physical activity levels (less than 150 min, 150 min (moderate), 150 min (vigorous)). Data on physical health were also collected.

Demographic data included age in years (18–24, 25–34, 35–40, 41 +), self‐reported weight and height and BMI (calculated; weight/height, kg/m^2^), classified according to the World Health Organization definitions: underweight (< 18.5 kg/m^2^); normal weight (18.5–24.9 kg/m^2^) (referred to as recommended BMI); overweight (25–29.9 kg/m^2^); and obese (≥ 30 kg/m^2^) [15]. Geographical location (country and city) was determined using anonymised IP address.

### Outcome measures

Outcome variables for the logistic regression were pregnancy intention (active planners or non-active planners) or length of time trying to conceive (active planners who had stopped contraception less than or more than a year ago).

### Data and statistical analysis

Duplicate entries were removed as identified by identical time, date, and anonymised IP address. Any biologically implausible BMI values were excluded (4.5% of respondents; height outside range 140-190 cm and weight outside range 30-190 kg) based on international weight and height data and a cohort of obese pregnant women [16–18]. The respondent’s location was determined from anonymised IP addresses which also enabled identification of non-UK resident women, who were excluded. As identified in the Tables, not all questions were answered by all women. The maximal number available for analysis is used for each question.

Descriptive statistics were calculated, with frequencies and percentages presented for categorical variables. Logistic regression was used to assess the association between each of the pregnancy planning outcome measures and health behaviours. Both adjusted and unadjusted odds ratios were calculated for each of the pregnancy planning measures. A Bonferroni correction was applied to adjust the p-values in the univariate logistic regression models to account for multiple testing and identify which variables were significant before proceeding to adjusted models. The multivariable regression models were adjusted for age, BMI, smoking, drug use, alcohol consumption, caffeine consumption, physical activity level, consumption of fruit and vegetables and folic acid supplementation. Health behaviours were also compared between those active planners who had stopped contraception more, or less than one year ago. Interaction effects between age and BMI and each of the health behaviours; smoking, drug use, alcohol consumption and folic acid supplementation were also examined. Statistical analysis was performed using RStudio.

## Results

### Study population

**Fig. 1:**
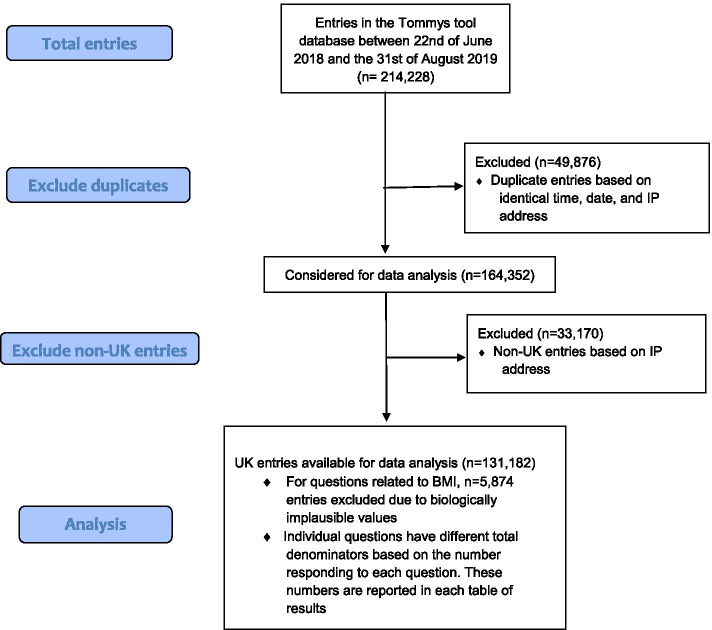
Flow diagram

**Fig. 2:**
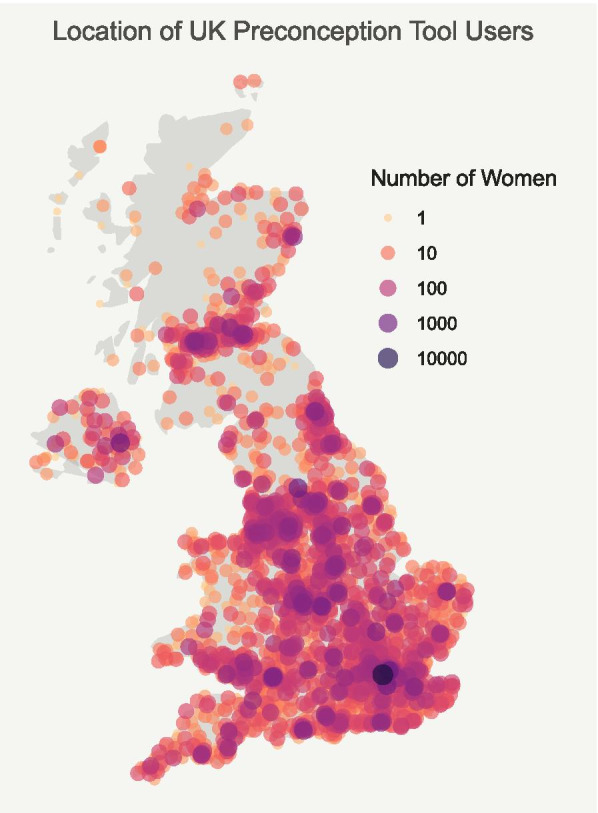
Map conveying the estimated number of users of the digital preconception tool throughout the UK from anonymised IP addresses

Between 22nd of June 2018 and the 31st of August 2019, 214,228 entries were recorded in the Tommy’s tool database (Fig. 1). After deduplication of entries based on time, date and IP address this reduced to 164,352 users, of whom 131,182 were in the UK, and geographically distributed across England, Wales, Scotland and Northern Ireland (Fig. 2). Of those with a known age (*n* = 127,102), 38% of women were aged 18–24 years, 51% were aged 25–34 years, 10% were aged 35–40 years and the remaining 1% were aged 41 years or above. For the women with height and weight data 5,874 were excluded due to implausible values, giving *n* = 111,032. Of this, 3% were underweight, 42% were of recommended BMI, 26% were overweight and 29% obese. Sixty-four point eight percent (*n* = 85,040 of 131,151 who responded) of women were actively planning for pregnancy while 35.2% (*n* = 46,111) were not actively planning. Of those who were active planners, 46.9% (*n* = 39,912) had stopped contraception over a year ago.

### Health behaviours and physical health

Twenty percent (*n* = 25,445) of women planning a pregnancy reported smoking and 3.7% (*n* = 4,698) reported illicit/recreational drug use. Alcohol consumption was reported by 54.5% (*n* = 69,195), while caffeine consumption was reported by 79.6% (*n* = 100,901). 42% of women (*n* = 49,072) participated in less than the recommended 150 min of weekly activity and just over half of women (53.3%, *n* = 61,747) reported consuming five portions of fruit and vegetables at least 4 days each week. Folic acid supplement use was low (31.5%, *n* = 31,329). 14.9% (*n* = 17,166) of women reported having a physical health condition or a condition in a previous pregnancy, of whom less than half (*n* = 6942, 40.6%) had spoken to a doctor or specialist about their plans for pregnancy (Table 1).

**Table 1:** Participant characteristics, health behaviours and numbers of women who provided a response

	**Number of women**	**Percentage of women**
**Age**		
18–24	47,977	37.8%
25–34	64,684	50.9%
35–40	12,905	10.2%
41 +	1,536	1.2%
Question not answered	4,080	-
**BMI**		
< 18.5	3,826	3.5%
18.5–24.9	46,739	42.1%
25–29	28,559	25.7%
30 +	31,908	28.7%
BMI excluded	5,874	-
Question not answered	14,276	-
**Smoking**		
No	101,611	80.0%
Yes	25,445	20.0%
Question not answered	4,126	-
**Illicit or recreational drug use**		
No	122,288	96.3%
Yes	4,698	3.7%
Question not answered	4,196	-
**Alcohol consumption**		
No	57,772	45.5%
Yes	69,195	54.5%
Question not answered	4,215	-
**Caffeine consumption**		
No	25,921	20.4%
Yes	100,901	79.6%
Question not answered	4,360	-
**Weekly level of activity**		
Less than 150 min of activity	49,072	42.0%
At least 150 min of moderate activity	50,813	43.5%
150 or more minutes of vigorous activity	16,948	14.5%
Question not answered	14,349	-
**Daily consumption of five portions of fruit and vegetables**		
No	36,590	31.6%
Yes	61,747	53.3%
Don’t know	17,463	15.1%
Question not answered	15,388	-
**Folic acid supplementation**		
No	68,033	68.5%
Yes	31,329	31.5%
Question not answered	31,820	-
**Physical health condition**^a^		
No	98,316	85.1%
Yes	17,166	14.9%
Question not answered	15,700	-
**Has a Physical health condition and consulted doctor or specialist about pregnancy plans**		
No	10,151	59.4%
Yes	6,942	40.6%
Question not answered	114,089	-

^a^This could include asthma, high blood pressure, heart problems, auto-immune conditions, acne, pre-eclampsia, gestational diabetes or epilepsy

### Women actively planning a pregnancy (not using contraception)

Women who took folic acid supplements were more likely to be active planners than those who did not take supplements (adjusted OR 7.27, 95% CI 6.97–7.59) with 44% of active planners taking supplements compared to 9.2% of non-active planners (Table 2). Folic acid supplementation was lower in active planners aged 18–24 years, of whom 29.1% (95% CI 28.9–29.3) reported taking supplements, almost half the percentage compared to older groups (Table 3). Folic acid supplementation was also lower in active planners who were underweight and obese where 36.9% (95% CI 36.2–37.6) and 39.3% (39.1–39.7) respectively reported taking supplements (Table 4).

**Table 2:** Participant health behaviours stratified by pregnancy intention

		**Percentage of women (95% CI)**			
	**Number of women**	Non-active planners (*n* = 46,111)	Active planners (*n* = 85,040)	Unadjusted OR (95% CI)	Adjusted OR (95% CI)^a^
**Age (*n* = 127,101)**					
18–24	47,977	50.4% (49.9–50.8)	30.7% (30.4–31.0)	1.00 (ref)	1.00 (ref)
25–34	64,683	43.8% (43.3–44.2)	54.8% (54.5–55.2)	2.06 (2.01–2.11)	1.82 (1.76–1.88)
35–40	12,905	5.3% (5.1–5.6)	12.8% (12.6–13.1)	3.94 (3.76–4.13)	3.40 (3.19–3.62)
41 +	1536	0.5% (0.4–0.6)	1.6% (1.5–1.7)	5.29 (4.59–6.10)	4.60 (3.84–5.50)
Question not answered	4081	-	-	-	-
**BMI (*n* = 111,031)**					
18.5–24.9 (Recommended)	46,739	45.6% (45.1–46.1)	40.2% (39.8–40.5)	1.00 (ref)	1.00 (ref)
< 18.5 (Underweight)	3826	3.8% (3.6–4.0)	3.2% (3.1–3.4)	0.96 (0.90–1.02)	1.05 (0.97–1.13)
25–29 (Overweight)	28,559	25.5% (25.1–25.9)	25.8% (25.5–26.2)	1.15 (1.12–1.19)	1.12 (1.08–1.16)
30 + (Obese)	31,907	25.1% (24.7–25.5)	30.8% (30.4–31.1)	1.39 (1.35–1.44)	1.48 (1.42–1.55)
Question not answered/BMI excluded	20,151	-	-	-	-
**Smoking (*n* = 127,056)**					
No	101,611	83.0% (82.7–83.3)	78.3% (78.0–78.6)	1.00 (ref)	1.00 (ref)
Yes	25,445	17.0% (16.7–17.3)	21.7% (21.4–22.0)	1.35 (1.31–1.39)	1.87 (1.79–1.94)
Question not answered	4126	-	-	-	-
**Illicit or recreational drug use (*n* = 126,985)**					
No	122,287	96.0% (95.8–96.1)	96.5% (96.4–96.6)	1.00 (ref)	1.00 (ref)
Yes	4698	4.0% (3.9–4.2)	3.5% (3.4–3.6)	0.86 (0.81–0.92)	0.99 (0.92–1.08)
Question not answered	4166	-	-	-	-
**Alcohol consumption (*n* = 126,966)**					
No	57,771	35.8% (35.3–36.2)	50.9% (50.5–51.2)	1.00 (ref)	1.00 (ref)
Yes	69,195	64.2% (63.8–64.7)	49.1% (48.8–49.5)	0.54 (0.53–0.55)	0.58 (0.56–0.60)
Question not answered	4185	-	-	-	-
**Caffeine consumption (*n* = 126,820)**					
No	25,919	19.1% (18.7–19.5)	21.2% (20.9–21.5)	1.00 (ref)	1.00 (ref)
Yes	100,901	80.9% (80.5–81.3)	78.8% (78.5–79.1)	0.88 (0.85–0.91)	1.00 (0.96–1.04)
Question not answered	4331	-	-	-	-
**Weekly level of activity (*n* = 116,832)**					
Less than 150 min of activity	49,071	40.9% (40.4–41.4)	42.6% (42.3–43.0)	1.00 (ref)	1.00 (ref)
At least 150 min of moderate activity	50,813	44.4% (43.9–44.9)	43.0% (42.6–43.4)	0.93 (0.91–0.95)	0.91 (0.88–0.94)
150 or more minutes of vigorous activity	16,948	14.7% (14.4–15.1)	14.4% (14.1–14.6)	0.94 (0.90–0.97)	0.94 (0.90–0.98)
Question not answered	14,319	-	-	-	-
**Daily consumption of five portions of fruit and vegetables (*n* = 115,793)**					
No	36,589	32.5% (32.0–32.9)	31.1% (30.8–31.4)	1.00 (ref)	1.00 (ref)
Yes	61,747	52.0% (51.5–52.5)	54.1% (53.7–54.4)	1.09 (1.06–1.12)	0.92 (0.89–0.95)
Don’t know	17,463	15.5% (15.2–15.9)	14.8% (14.6–15.1)	1.00 (0.96–1.04)	1.05 (1.00–1.10)
Question not answered	15,352	-	-	-	-
**Folic acid supplementation (*n* = 99,362)**					
No	68,033	90.8% (90.5–91.1)	56.0% (55.6–56.3)	1.00 (ref)	1.00 (ref)
Yes	31,329	9.2% (8.9–9.5)	44.0% (43.7–44.4)	7.75 (7.45–8.06)	7.27 (6.97–7.59)
Question not answered	31,789	-	-	-	-

^a^ models adjusted for age, BMI, smoking, drug use, alcohol consumption, caffeine consumption, activity level, consumption of five-a-day & folic acid supplementation

**Table 3:** Health behaviours of women actively planning pregnancy (not using contraception) stratified by age

		**Percentage of active planners (95% CI)**			
		Age			
	**Number of active planners**	18–24 (*n* = 25,086)	25–34 (*n* = 44,795)	35–40 (*n* = 10,479)	41 + (*n* = 1310)
**Smoking (*n* = 81,118)**					
No	63,561	69.1% (68.5–69.6)	82.2% (82.1–82.3)	83.2% (82.9–83.4)	85.0% (84.3–85.7)
Yes	17,557	30.9% (30.4–31.5)	17.8% (17.7–17.9)	16.9% (16.6–17.1)	15.0% (14.3–15.7)
Question not answered	552	-	-	-	-
**Illicit or recreational drug use (*n* = 81,145)**					
No	78,301	94.7% (94.6–94.8)	97.3% (97.2–97.3)	97.4% (97.3–97.5)	96.8% (96.5–97.1)
Yes	2844	5.3% (5.2–5.4)	2.7% (2.7–2.8)	2.6% (2.5–2.7)	3.2% (2.9–3.5)
Question not answered	525	-	-	-	-
**Alcohol consumption (*n* = 81,128)**					
No	41,252	57.0% (56.8–57.2)	47.5% (47.4–47.7)	49.4% (49.1–49.7)	57.1% (56.2–58.0)
Yes	39,876	43.0% (42.8–43.2)	52.5% (52.3–52.6)	50.6% (50.3–50.9)	42.9% (42.0–43.8)
Question not answered	542	-	-	-	-
**Caffeine consumption (*n* = 81,029)**					
No	17,154	23.4% (23.3–23.6)	19.8% (19.7–19.9)	21.1% (20.8–21.4)	25.7% (24.9–26.5)
Yes	63,875	76.6% (76.4–76.8)	80.2% (80.1–80.3)	78.9% (78.6–79.2)	74.3% (73.5–75.1)
Question not answered	641	-	-	-	-
**Weekly level of activity (*n* = 74,600)**					
Less than 150 min of activity	31,783	42.4% (42.2–42.7)	43.0% (42.8–43.2)	41.9% (41.5–42.2)	38.0% (37.0–38.9)
At least 150 min of moderate activity	32,097	41.8% (41.6–42.1)	43.6% (43.4–43.8)	43.1% (42.8–43.5)	44.3% (43.3–45.2)
150 or more minutes of vigorous activity	10,720	15.7% (15.6–15.9)	13.4% (13.3–13.5)	15.0% (14.8–15.2)	17.8% (17.1–18.5)
Question not answered	7070	-	-	-	-
**Daily consumption of five portions of fruit and vegetables (*n* = 74,050)**					
No	23,031	36.0% (35.7–36.2)	30.2% (30.0–30.3)	25.0% (24.7–25.3)	22.6% (21.8–23.4)
Yes	40,032	43.7% (43.4–43.9)	56.7% (56.5–56.9)	64.6% (64.3–64.9)	69.2% (68.3–70.0)
Don’t know	10,987	20.4% (20.2–20.6)	13.1% (13.0–13.2)	10.4% (10.2–10.6)	8.2% (7.7–8.8)
Question not answered	7620	-	-	-	-
**Folic acid supplementation (*n* = 63,160)**					
No	35,333	70.8% (70.7–71.1)	50.9% (50.7–51.1)	44.5% (44.1–44.8)	45.5% (44.5–46.5)
Yes	27,827	29.1% (28.9–29.3)	49.1% (48.9–49.3)	55.5% (55.2–55.9)	54.5% (53.5–55.5)
Question not answered	18,510	-	-	-	-

**Table 4:** health behaviours of women actively planning pregnancy stratified by BMI

		**Percentage of active planners (95% CI)**			
		BMI			
	**Number of active planners**	< 18.5 (*n* = 2311)	18.5–24.9 (*n* = 28,710)	25–29 (*n* = 18,473)	30 + (*n* = 21,990)
**Smoking (*n* = 71,116)**					
No	56,301	68.7% (68.0–69.3)	81.4% (81.3–81.6)	80.2% (80.0–80.4)	76.5% (76.3–76.7)
Yes	14,815	31.3% (30.7–32.0)	18.6% (18.4–18.7)	19.8% (19.6–20.0)	23.5% (23.3–23.7)
Question not answered	368	-	-	-	-
**Illicit or recreational drug use (*n* = 71,328)**					
No	68,934	94.6% (94.3–94.9)	96.2% (96.1–96.3)	97.1% (97.0–97.2)	97.0% (96.9–97.1)
Yes	2394	5.4% (5.1–5.7)	3.8% (3.7–3.9)	2.9% (2.8–3.0)	3.0% (2.9–3.1)
Question not answered	156	-	-	-	-
**Alcohol consumption (*n* = 71,381)**					
No	35,428	61.0% (60.3–61.7)	46.0% (45.8–46.2)	47.5% (47.2–47.7)	55.0% (54.7–55.2)
Yes	35,953	39.0% (38.3–39.7)	54.0% (53.8–54.2)	52.5% (52.3–52.8)	45.0% (44.8–45.3)
Question not answered	103	-	-	-	-
**Caffeine consumption (*n* = 71,393)**					
No	14,621	22.8% (22.2–23.4)	20.1% (19.9–20.2)	19.9% (19.7–20.1)	21.3% (21.1–21.4)
Yes	56,772	77.2% (76.6–77.8)	79.9% (79.8–80.1)	80.1% (79.9–80.3)	78.7% (78.6–78.9)
Question not answered	91	-	-	-	-
**Weekly level of activity (*n* = 71,224)**					
Less than 150 min of activity	30,261	40.9% (40.2–41.6)	38.4% (38.2–38.6)	42.7% (42.4–42.9)	47.8% (47.6–48.0)
At least 150 min of moderate activity	31,041	43.5% (42.8–44.2)	45.8% (45.6–46.0)	43.9% (43.7–44.2)	40.4% (40.2–40.7)
150 or more minutes of vigorous activity	9922	15.6% (15.1–16.1)	15.8% (15.7–16.0)	13.4% (13.2–13.6)	11.7% (11.6–11.9)
Question not answered	260	-	-	-	-
**Daily consumption of five portions of fruit and vegetables (*n* = 70,602)**					
No	21,864	36.4% (35.7–37.1)	30.1% (29.9–30.3)	30.5% (30.3–30.7)	31.9% (31.7–32.1)
Yes	38,307	46.8% (46.1–47.5)	55.4% (55.2–55.6)	54.9% (54.7–55.2)	53.0% (52.8–53.2)
Don’t know	10,431	16.8% (16.3–17.4)	14.5% (14.3–14.6)	14.6% (14.4–14.8)	15.1% (15.0–15.3)
Question not answered	882	-	-	-	-
**Folic acid supplementation (*n* = 60,318)**					
No	33,487	63.1% (62.4–63.8)	53.3% (53.1–53.5)	54.7% (54.4–54.9)	60.6% (60.3–60.9)
Yes	26,831	36.9% (36.2–37.6)	46.7% (46.5–46.9)	45.3% (45.1–45.6)	39.3% (39.1–39.7)
Question not answered	11,166	-	-	-	-

Smokers were more likely to be active planners compared to non-smokers (adjusted OR 1.87, 95% CI 1.79–1.94) with 21.7% (95% CI 21.4–22.0) of active planners reporting smoking (Table 2). The youngest group of active planners were the most likely to smoke; 31% compared to 15–18% in the older age groups (Table 3). There was also a high frequency of smoking amongst active planners who were underweight, 31.3% (95% CI 30.7–32.0) (Table 4).

Women who consumed alcohol were less likely to be active planners (adjusted OR 0.58, 95% CI 0.56–0.60). Illicit or recreational drug use was most common in the active planners in the 18–24 age group and in those who were underweight, with a similar frequency in both groups (~ 5%). Albeit statistically significant, differences in other health behaviours were relatively small between active and non-active planners.

Active planners were twice as likely to have stopped contraception over a year ago if they were aged between 35–40 years (adjusted OR 2.17, 95% CI 2.05–2.30) and almost 3 times as likely if they were aged 41 and above (adjusted OR 2.88, 95% CI 2.52–3.29), compared to those in the 18–24 age group. Women in the underweight (adjusted OR 1.19, 95% CI 1.09–1.29) and obese (adjusted OR 1.30, 95% CI 1.24–1.36) BMI groups were also more likely to have stopped contraception over a year ago than those in the recommended BMI group. Active planners who smoked and used illicit/recreational drugs were more likely to have stopped contraception over a year ago (adjusted OR 1.25, 95% CI 1.20–1.31 and adjusted OR 1.17, 95% CI 1.07–1.28, respectively). In this group of active planners, drinking alcohol and taking folic acid was less likely in those who had stopped contraception over a year ago (adjusted OR 0.74, 95% CI 0.72–0.77 and adjusted OR 0.88, 95% CI 0.85–0.91, respectively) (Table 5).

**Table 5:** Participant health behaviours stratified by duration of pregnancy planning

		**Percentage of active planners (95% CI)**			
	**Number of active planners**	Stopped contraception < 1 year ago (*n* = 42,961)	Stopped contraception > 1 year ago (*n* = 39,912)	Unadjusted OR (95% CI)	Adjusted OR^a^ (95% CI)
**Age (*n* = 81,533)**					
18–24	25,038	33.2% (32.7–33.6)	28.0% (27.6–28.5)	1.00 (ref)	1.00 (ref)
25–34	44,732	55.9% (55.4–56.4)	53.8% (53.3–54.3)	1.14 (1.10–1.18)	1.27 (1.22–1.32)
35–40	10,456	9.9% (9.6–10.2)	16.0% (15.6–16.4)	1.91 (1.83–2.00)	2.17 (2.05–2.30)
41 +	1307	1.0% (0.9–1.1)	2.2% (2.1–2.4)	2.53 (2.25–2.85)	2.88 (2.52–3.29)
Question not answered	1340	-	-	-	-
**BMI (*n* = 71,373)**					
18.5–24.9 (Recommended)	28,662	42.4% (41.9–42.9)	37.7% (37.2–38.3)	1.00 (ref)	1.00 (ref)
< 18.5 (Underweight)	2304	3.1% (2.9–3.3)	3.4% (3.2–3.5)	1.21 (1.11–1.32)	1.19 (1.09–1.29)
25–29 (Overweight)	18,443	26.6% (26.1–27.0)	25.0% (24.6–25.5)	1.06 (1.02–1.10)	1.04 (1.00–1.08)
30 + (Obese)	21,964	27.9 (27.5–28.4)	33.9% (33.4–34.4)	1.36 (1.32–1.41)	1.30 (1.24–1.36)
Question not answered/BMI excluded	11,500	-	-	-	-
**Smoking (*n* = 81,737)**					
No	64,014	80.3% (79.9–80.6)	76.2% (75.8–76.6)	1.00 (ref)	1.00 (ref)
Yes	17,723	19.7% (19.4–20.1)	23.8% (23.4–24.2)	1.27 (1.23–1.31)	1.25 (1.20–1.31)
Question not answered	1136	-	-	-	-
**Illicit or recreational drug use (*n* = 81,678)**					
No	78,813	96.8% (96.6–96.9)	96.2% (96.0–96.4)	1.00 (ref)	1.00 (ref)
Yes	2865	3.2% (3.1–3.4)	3.8% (3.6–4.0)	1.18 (1.09–1.27)	1.17 (1.07–1.28)
Question not answered	1195	-	-	-	-
**Alcohol consumption (*n* = 81,659)**					
No	41,532	47.5% (47.1–48.0)	54.4% (53.9–54.9)	1.00 (ref)	1.00 (ref)
Yes	40,127	52.5% (52.0–52.9)	45.6% (45.1–46.1)	0.76 (0.74–0.78)	0.74 (0.72–0.77)
Question not answered	1214	-	-	-	-
**Caffeine consumption (*n* = 81,568)**					
No	17,278	20.4% (20.0–20.8)	22.0% (21.6–22.4)	1.00 (ref)	1.00 (ref)
Yes	64,290	79.6% (79.2–80.0)	78.0% (77.6–78.4)	0.91 (0.88–0.94)	0.94 (0.90–0.98)
Question not answered	1305	-	-	-	-
**Weekly level of activity (*n* = 75,076)**					
Less than 150 min of activity	32,002	42.9% (42.4–43.3)	42.4% (41.9–42.9)	1.00 (ref)	1.00 (ref)
At least 150 min of moderate activity	32,291	43.1% (42.6–43.6)	43.0% (42.4–43.5)	1.01 (0.98–1.04)	1.03 (1.00–1.07)
150 or more minutes of vigorous activity	10,783	14.1% (13.7–14.4)	14.7% (14.3–15.0)	1.05 (1.01–1.10)	1.08 (1.02–1.13)
Question not answered	7797	-	-	-	-
**Daily consumption of five portions of fruit and vegetables (*n* = 74,403)**					
No	23,144	30.7% (30.2–31.1)	31.6% (31.1–32.1)	1.00 (ref)	1.00 (ref)
Yes	40,216	54.8% (54.3–55.3)	53.2% (32.7–53.7)	0.94 (0.91–0.97)	0.93 (0.90–0.97)
Don’t know	11,043	14.5% (14.2–14.9)	15.2% (14.8–15.6)	1.02 (0.97–1.06)	1.03 (0.98–1.09)
Question not answered	8470	-	-	-	-
**Folic acid supplementation (*n* = 63,541)**					
No	35,561	54.9% (54.4–55.5)	57.1% (56.5–57.7)	1.00 (ref)	1.00 (ref)
Yes	27,980	45.1% (44.5–45.6)	42.9% (42.3–43.5)	0.92 (0.89–0.95)	0.88 (0.85–0.91)
Question not answered	19,332	-	-	-	-

^a^ models adjusted for age, BMI, smoking, drug use, alcohol consumption, caffeine consumption, activity level, consumption of five-a-day & folic acid supplementation

## Discussion

### Main findings and interpretation

We have examined the health behaviours and lifestyle factors of the largest cohort to date of UK women planning a pregnancy. Given that there are approximately 650,000 births in England and Wales every year [19], and the wide geographical distribution of the respondents, this survey is likely to reflect the behaviours of a large proportion of UK women planning pregnancy. Our analysis indicates that many health behaviours were sub-optimal including smoking, lack of folic acid supplementation, alcohol consumption, low levels of physical activity and of fruit and vegetable intake in the diet. These unhealthy behaviours were most evident in younger women and in those who were underweight. A shift towards healthier behaviours was observed in women actively planning pregnancy, except for smoking, however this improvement appears transient, as evidenced by poorer health behaviours in women trying to conceive for more than a year. Together, these data show that UK women, over half of whom plan pregnancy (54%) [20], are not adequately prepared, which has broad implications for the health of this and future generations.

We found that approximately one in five women reported smoking, a similar proportion to a retrospective UK study in 2014 of 1173 women which found that 21% of women smoked [7] and in alignment with national data from 2019 which reported 21.2% in women aged 25–34 years [21]. Our finding that smokers were more likely to be actively trying to conceive than non-smokers was unexpected and contrasts with a recent report (2020) of 294 women in Australia where no difference was found between these two groups [3]. The observation that smoking was most prevalent in those who stopped contraception greater than one year might reflect lack of sustained change in behaviour after a year of failing to conceive. It alternatively reflects more difficulty in conceiving in those with poorer health behaviours.

Despite widespread evidence-based public health guidance on the benefits of taking folic acid in the preconception period, only one in three women planning pregnancy reported supplement use. Women taking supplements were more likely to be active planners, although more than half of this group i.e. women who had stopped contraception, took no folic acid. Clearly, the recommendation that all women planning pregnancy should take folic acid remains unheeded by many. Little has changed in the past two decades; in a UK survey undertaken between 1998 and 2002, 44% of women who became pregnant within three months of participating had taken folic acid supplements [22]. This sustained lack of adherence to public health advice amongst those planning pregnancy, notwithstanding unintended pregnancies, strongly supports mandatory folic acid fortification in the UK. Estimates suggest that around 2000 pregnancies affected by neural tube defects between 1998 and 2012 would have been prevented if fortification had been in place [23]. In June 2019, the UK government issued a public consultation on mandatory fortification with a set of questions on its effectiveness and feasibility [24], the outcome of which is awaited with interest.

Amongst the respondents, nearly 50% were overweight or obese which is similar to UK women of reproductive age. Obesity affects oocyte function, early embryonic development, fetal growth and complications in pregnancy and at delivery [4]. In addition, sub-optimal nutrition before pregnancy may adversely impact pregnancy outcomes by increasing the risk of micronutrient deficiencies implicated in fetal growth and development [1]. Furthermore, inadequate physical activity before pregnancy may increase the risk of gestational diabetes and pre-eclampsia [25].

Given that many pre-existing health conditions, as well as health problems in a previous pregnancy increase the risk of adverse pregnancy outcome, it was notable that of the 15% of women declaring ongoing health conditions or health issues in a previous pregnancy, only half had sought clinical advice before considering pregnancy.

It is of little surprise that increasing age, and underweight and obese BMI were associated with reduced fertility, further emphasising the need for targeted health messaging and intervention.

High-risk behaviours were common in younger women; this was illustrated by a high prevalence of smoking and drug use as well as a lack of folic acid supplementation in the 18 to 24 age group actively planning pregnancy, and aligns with a US study (*n* = 847) which observed a high prevalence of long term binge drinking and smoking in this age group [26]. A systematic review also reported that younger women were less aware of the importance of folic acid supplementation [27], and as evidenced by analysis of hair samples, younger women are more likely to use recreational drugs in the periconceptual period [28]. The Preconception Partnership’s proposal for preconception health highlighted the presence of social and medical risks in younger women and a need for more effective methods to engage and support this group [2].

### Strengths and limitations

Our study has a number of strengths. It is the largest sample of health behaviours and lifestyle factors in women planning a pregnancy studied to date, and the breadth of questions provides a wealth of information. A high proportion of the women who used the digital preconception tool completed the questionnaire, perhaps reflecting the user friendliness of the online platform. Studies with a self-selected sample such as this, are recognised to be subject to social desirability bias where high-risk health behaviours may be underreported [7]. However, the high prevalence of these behaviours, and the wide geographical distribution suggests that the tool was successful in targeting harder to reach groups.

We acknowledge several limitations. The brevity of the questionnaire, whilst likely to be instrumental in the wide response, inevitably restricted the scope of analysis. Information on socio-economic status and ethnicity was lacking. When reporting health behaviours such as drinking alcohol, women were not asked the frequency of consumption which would have enriched data interpretation. There may also be an element of volunteer bias to the responses, as those using the digital tool were likely to include a subgroup actively seeking advice on their preconception health behaviours, and therefore may not be representative of *all* UK women planning a pregnancy. Anonymised IP addresses are associated with some inaccuracy when identifying location. Lastly, an assumption was made that women utilising the tool were planning a pregnancy.

## Conclusion

This unique study presents health behaviours of the largest group of women planning a pregnancy to date, including a novel insight into behaviours amongst women trying to conceive for less than, and over one year. We have clearly shown that women are not adequately preparing for pregnancy, evidenced by a high prevalence of smoking, low folic acid use, low levels of physical activity and inadequate fruit and vegetable intake. Smoking and lack of folic acid were particularly common in both younger women and underweight women and in those trying to conceive for over a year. Underlying medical conditions were common yet clinical advice rarely sought. This study highlights the importance of targeted support for such women planning pregnancy. Data from this study will contribute metrics for public health planning as suggested by the Preconception Partnership [2].

## Data Availability

The datasets used and/or analysed during the current study are available from the corresponding author on reasonable request.

## Abbreviations

PHE: Public Health England
RCOG: Royal College of Obstetricians and Gynaecologists
